# Frailty in end-stage hip or knee osteoarthritis: validation of the Groningen Frailty Indicator (GFI) questionnaire

**DOI:** 10.1007/s00296-017-3868-1

**Published:** 2017-11-17

**Authors:** Jennifer M. T. A. Meessen, Claudia S. Leichtenberg, Claire Tilbury, Bart L. Kaptein, Lennard A. Koster, P. Eline Slagboom, Suzan H. M. Verdegaal, Ron Onstenk, Henrike M. J. van der Linden-van der Zwaag, Herman Kaptijn, Stephan B. W. Vehmeijer, Willem-Jan C. Marijnissen, Pieter-Jan Damen, Rob G. H. H. Nelissen, Thea P. M. Vliet Vlieland

**Affiliations:** 1 Department of Orthopedics, Leids Universitair Medisch Centrum (LUMC), Leiden, The Netherlands; 2 Department of Molecular Epidemiology, Leids Universitair Medisch Centrum (LUMC), Leiden, The Netherlands; 3 Department of Orthopedics, Alrijne Ziekenhuis, Leiderdorp, The Netherlands; 4 Department of Orthopedics, Groene Hart Ziekenhuis, Gouda, The Netherlands; 5 Department of Orthopedics, Lange Land Ziekenhuis, Zoetermeer, The Netherlands; 6 Department of Orthopedics, Reinier de Graaf Gasthuis, Delft, The Netherlands; 7 Department of Orthopedics, Albert Schweitzer Ziekenhuis, Dordrecht, The Netherlands; 8 Department of Orthopedics, Waterland Ziekenhuis, Purmerend, The Netherlands

**Keywords:** Frailty, Osteoarthritis, Arthroplasty, Validation, Groningen Frailty Indicator

## Abstract

Frailty is highly prevalent in the elderly, increasing the risk of poor health outcomes. The Groningen Frailty Indicator (GFI) is a 15-item validated questionnaire for the elderly. Its value in patients with end-stage hip or knee osteoarthritis (OA) has not yet been determined. This study assesses the validity of the GFI in this patient group. End-stage hip or knee OA patients completed the GFI (range 0–15, ≥ 4 = frail) before arthroplasty surgery. Convergent validity was determined by Spearman-rank correlation between the SF-12 physical (PCS) and mental (MCS) component scores and the physical and mental GFI-domains, respectively. Discriminant validity was assessed by means of overall GFI-score and the pain-domain of the Hip/Knee Osteoarthritis Outcome Score (HOOS/KOOS). Altogether 3275 patients were included of whom 2957 (90.3%) completed the GFI. Mean GFI-scores were 2.78 (2.41) and 2.28 (1.99) in hip and knee OA-patients, respectively, with 570 (35.9%) of hip and 344 (24.1%) of knee patients considered frail. The convergent validity was moderate to strong (physical domain R = − 0.4, mental domain R = − 0.6) and discriminant validity low (R HOOS/KOOS-pain domain = − 0.2), confirming the validity of the GFI-questionnaire in this population. With 90% of participants completing the GFI, it is a feasible and valid questionnaire to assess frailty in end-stage hip and knee OA-patients. One-third (33.3%) of the patients undergoing hip arthroplasty and a quarter (24.1%) of those undergoing knee arthroplasty are frail. Whether this is associated with worse outcomes and can thus be used as a pre-operative predictor needs to be explored.

## Introduction

Osteoarthritis (OA) is a degenerative joint disease which often leads to disability and pain. A highly effective treatment for end-stage OA is arthroplasty surgery [1, 2]. Over 202,500 total hip and 402,100 total knee arthroplasties (THA and TKA) are performed annually in the United States of America alone [3], with the volume expected to increase up to sixfold by 2030 [3].

At present, 83% of the patients receiving THA and 79% of patients receiving TKA are older than 60 years of age [4]. As frailty is highly prevalent in the elderly, it is likely that a considerable proportion of patients undergoing THA or TKA are frail [5]. Although there is not one definition for frailty, the most often used definitions include a combination of decrease of independence, strength, cognition, activity, energy, weight and walking speed [6–12]. Literature shows that there is considerable heterogeneity in the extent of frailty individuals may experience, with some persons accelerating fast while others are slowly progressing to higher levels of frailty [13]. Within persons of the same age, also the onset of frailty differs per individual [14–17].

It is generally acknowledged that frailty hampers the ability to resist stressors, leading to vulnerability for adverse outcomes after surgery [6, 16–19]. As such, it is of importance to have more insight into frailty in the group of patients undergoing THA or TKA. As a first step into the exploration of the role of frailty in the outcomes of total joint surgery, an appropriate instrument for frailty is needed.

The Groningen Frailty Indicator (GFI) is a frequently used questionnaire in the elderly to assess frailty. The advantage of the GFI is that it is a self-reported score; furthermore, this questionnaire has been validated specifically for elderly (mean age 81 years). In these elderly (both community dwelling and institutionalized), it was found that the GFI is feasible, reliable and valid [20].

However, it is not known yet how feasible the GFI is in a clinical setting as well as the validity of the GFI amongst the somewhat younger patients with end-stage hip or knee OA waiting for arthroplasty surgery.

Therefore, in this study we aimed to assess the feasibility and validity of the GFI as a tool to measure frailty in end-stage hip or knee osteoarthritis patients scheduled to undergo arthroplasty surgery.

## Methods

### Study design

This study is part of the Longitudinal Leiden Orthopaedics Outcomes of Osteo-Arthritis study (LOAS). The LOAS study is an ongoing, multi-center, longitudinal prospective cohort study including patients undergoing primary total hip or knee arthroplasty (THA or TKA). Participants are recruited in 7 participating hospitals (the Leiden University Medical Center, Leiden; Alrijne Hospital, Leiden/Leiderdorp (former Diaconessenhuis and Rijnland Hospital); Groene Hart Hospital, Gouda; LangeLand Hospital, Zoetermeer; Reinier de Graaf Gasthuis, Delft; Albert Schweitzer Hospital, Dordrecht; Waterland Hospital, Purmerend). The LOAS study (Trial ID NTR3348) started in June 2012. The present study is only concerned with data gathered preoperatively from June 2012 to June 2016 [21].

### Patients

All patients who were able to complete questionnaires in Dutch and who were 18 years or older were eligible for participation. Excluded were patients who did not provide informed consent, had insufficient Dutch language skills or of whom the physical or mental status did not allow participation. Eligible patients were informed about the study through written and oral information by their treating surgeon at the outpatient clinic. Only patients who agreed to be approached by the researcher received additional written information about the study by regular mail or e-mail, as well as a questionnaire, a stamped return envelope and a consent form. Patients were included in the study once written informed consent was obtained according to the Declaration of Helsinki [22]. For the purpose of the present analysis only data from patients who returned the preoperative questionnaire between the start of the study in June 2012 until June 2016 were included. Ethical approval was obtained by the Medial Ethics Committee of the Leiden University Medical Center (registration number P12.047) and funding was received from the Dutch Arthritis Foundation (LLP13).

The questionnaires were incorporated in current clinical setting of the included hospitals which all participate in the collection of patient reported outcome measures (PROMs) for the national Dutch Arthroplasty Register (LROI).

### Assessments

#### Frailty

Frailty was assessed by the Groningen Frailty Indicator (GFI). This questionnaire consists of 15 questions covering several aspects of life, such as independence in daily tasks, involuntary weight loss, medication use, mental state, vision and hearing. Together these questions lead to a score between 0 and 15, a score of ≥ 4 is considered to be frail. The GFI is specifically directed to elderly persons both living at home as well as in institutions [20, 23, 24].

#### Overall health

Quality of life was measured using the validated Dutch version of the Short Form (SF)-12 [25]. The SF-12 comprises 12 items on generic measurement of the overall health-related quality of life. Scores range from 0 to 100, with 0 being lowest possible score and 100 the highest. From the SF-12, two subscales can be calculated, the physical component score (PCS) and mental component score (MCS). These subdomains were assessed separately in the analyses [26].

#### Hip/knee symptoms

The hip disability/knee injury and osteoarthritis outcome score (HOOS/KOOS) questionnaires are validated questionnaires to measure the function of patients with end-stage osteoarthritis for hip or knee, respectively [27, 28]. These questionnaires comprise five domains (activities of daily living, quality of life, sports, symptoms and pain). For the current study the validated Dutch version was used [29, 30].

### Statistical analyses

Patient characteristics were analysed using descriptive statistics. Rates of patients who did not, partially or completely fill out the GFI were computed. Comparisons between patients who filled in the GFI completely and those who did not or partially were done by means of either Chi-square tests for categorical variables and *t* tests for continuous variables. In addition, for each GFI item the proportion of missing values was determined.

To explore determinants for completing the questionnaire a binary variable “completion of questionnaire” was constructed. This variable was used in a logistic regression analysis to see if age, sex, BMI and comorbidities are of significant influence on the completion of the questionnaire.

The internal consistency of the GFI in this patient population was assessed by means of Cronbach’s alpha, with an alpha of > 0.7 being considered as good consistency [31]. Convergent validity of the GFI was determined by computing correlations between the physical domain of GFI (questions 1–9) and the PCS of the SF-12. The mental domain of the GFI (question 14 and 15) was correlated with the MCS of the SF-12. Correlations were computed using a Spearman rank correlation coefficients. As the corresponding subscales of the GFI and SF-12 aim to measure similar constructs it was hypothesized that the correlation between the subscales of the GFI and SF-12 will be high.

Discriminant validity of the questionnaire was assessed by correlating the physical domain of the GFI to the MCS and the mental domain of the GFI to the PCS. Also, a spearman rank correlation analysis including the total GFI-score and pain as measured by the HOOS/KOOS questionnaire was performed. As the correlated constructs are conceptually different, we hypothesized the correlation between these domains would be low.

For those THA and TKA patients who completed the GFI the prevalence of frailty was calculated, based on the cut-off score of four [24]. The demographic variables of those assigned frail and those not designated as frail were compared by means of a *t* test or Chi-square test, whichever was appropriate. All analyses were performed with IBM SPSS statistics software version 23.

## Results

Within the time frame of the present analysis 3275 patients with end-stage hip OA (*N* = 1691) and knee OA (*N* = 1584) were included in the cohort study. For both end-stage hip and knee OA, 90.3% of the participants completed the questionnaire. In Table 1 the socio-demographic variables of patients returning the questionnaire that did and did not complete it fully were compared. In hip OA, those who did not fully complete the questionnaire were significantly older, whereas in knee OA those who did not complete the questionnaire fully were more often female and had a lower score on the HOOS/KOOS-activities of daily life domain. In both end-stage hip and knee OA those who did not complete the questionnaire had a significantly lower score on the MCS.

**Table 1:** Characteristics of patients with end-stage hip or knee osteoarthritis undergoing total hip or knee arthroplasty who did and did not complete the Groningen Frailty Indicator questionnaire (GFI)

			Hip					Knee				
			GFI fully completed *N* = 1527		GFI not completed *N* = 164		*P* value*	GFI fully completed *N* = 1430		GFI not completed *N* = 154		*P* value*
Sex	Female	*N* (%)	925	61.5%	107	67.3%	0.155	911	64.2%	119	77.3%	0.001
Age	Years	Mean (SD)	67.8	9.8	70.9	9.4	< 0.001	67.4	8.9	67.6	9.1	0.818
BMI		Mean (SD)	27.2	4.3	27.0	5.4	0.529	29.4	4.7	29.0	4.4	0.373
Living	Not alone	*N* (%)	1187	77.7%	118	71.9%	0.097	1095	76.5%	115	75.7%	0.598
Comorbidity	Musculoskeletal	*N* (%)	259	17.8%	29	20.9%	0.370	326	24.1%	39	26.5%	0.522
	Other	*N* (%)	942	70.7%	80	69.0%	0.692	900	74.7%	85	73.9%	0.855
SF-12	PCS	Mean (SD)	32.2	9.4	32.4	9.2	0.821	32.3	9.1	32.4	9.7	0.918
	MCS	Mean (SD)	54.8	9.9	52.9	10.4	0.046	55.6	9.4	54.0	9.0	0.009
HOOS KOOS	Pain	Mean (SD)	37.9	18.6	39.8	20.0	0.244	38.9	17.6	36.4	18.8	0.124
	Symptoms	Mean (SD)	39.8	18.5	41.9	20.6	0.252	43.7	13.5	42.0	12.4	0.178
	Activities of daily life	Mean (SD)	39.9	19.2	41.8	21.6	0.324	45.0	18.2	40.8	20.9	0.026
	Sport	Mean (SD)	18.1	18.4	21.6	21.7	0.200	10.7	14.3	11.2	15.5	0.852
	Quality of life	Mean (SD)	33.4	10.8	35.2	12.1	0.083	33.6	10.4	34.6	11.8	0.327

*BMI* body mass index, *SF-12* short form 12 questionnaire, *PCS* physical component score of the SF-12, *MCS* mental component score of the SF-12, *HOOS/KOOS* hip disability/knee injury and osteoarthritis outcome score

* Characteristics of patients who completed and those who did not complete the GFI questionnaires were tested by means of a *t* test (normal distribution, continue), Mann–Whitney (not-normal distribution, continue) or Chi square (discrete) variables

On a total of 15 items, the median number of missing items for both joint locations was 0 (range 0 to 15), whereas the mean (SD) was 0.4 (1.9) (hip OA: 0.4 (2.0), knee OA: 0.3 (1.8)).

Of the 164 patients with hip OA who did not complete all questions, 29 did not fill in any question whereas 99 missed only one question. Of the 154 patients with knee OA who did not complete all questions, 21 did not fill in any question and 102 persons had only one missing question.

Table 2 shows the percentage of missing values per question. Most frequently missed was question 15 “*How would you rate your physical fitness on a scale of 1 to 10?*” for both hip and knee (hip 4.4% missing, knee 4.2% missing). This was the only question with no predefined answering options; instead patients had to write down the number themselves. In addition, in patients with hip OA question 2 *“Are you able to walk independently outside?”* (2.8% missing) and question 3 *“Are you able to (un)dress yourself?”* (2.7% missing) were relatively often missing, while in knee OA patients question 6 *“Do you encounter problems in daily life because of impaired hearing?”* (2.6% missing) and question 2 *“Are you able to walk independently outside?”* (2.3% missing) were relatively often missing.

**Table 2:** Percentage of missing per question for the Groningen Frailty Indicator

		Hip (%)	Knee (%)
1.	Are you able to do groceries by yourself?	2.5	1.9
2.	Are you able to walk independently outside?	2.8	2.3
3.	Are you able to (un)dress yourself?	2.7	2.2
4.	Are you able to use the bathroom by yourself?	2.7	2.0
5.	Do you encounter problems in daily life because of impaired vision?	2.5	2.1
6.	Do you encounter problems in daily life because of impaired hearing?	2.4	2.6
7.	Did you unintentionally lose weight over the past 6 months?	2.4	1.8
8.	Do you use 4 or more types of medication?	2.7	1.8
9.	Do you have any complaints on your memory?	2.1	1.8
10.	Do you experience emptiness around you?	2.2	1.8
11.	Do you miss the presence of other people around you?	2.4	2.0
12.	Do you feel left alone?	2.7	1.8
13.	Have you felt down or depressed lately?	2.5	2.0
14.	Have you felt nervous or anxious lately?	2.5	2.0
15.	How would you rate your physical fitness on a scale of 1 to 10?	4.4	4.2

To assess determinants for completing the GFI questionnaire a logistic regression model was build including age, sex, BMI, musculoskeletal and other comorbidities. Table 3 shows the odds ratios associated with this model. It was found that age and sex are statistically significant determinants for completing the questionnaire in persons with end-stage OA of the lower limb corrected for BMI and comorbidities.

**Table 3:** Odds ratios for demographic characteristics to completing the Groningen Frailty Questionnaire

	Odds ratio	95% confidence interval	*P* value
Age	0.981	0.966–0.997	0.020
Sex	1.497	1.100–2.038	0.010
Body mass index (BMI)	1.006	0.974–1.039	0.714
Musculoskeletal comorbidities	0.946	0.661–1.354	0.762
Other comorbidities	0.890	0.644–1.230	0.481

Characteristics were included in logistic regression analysis relating the demographic characteristics to completing the GFI questionnaire (yes/no)

Older age is, independent of gender, BMI and comorbidities, associated with lower odds for completing the questionnaire (OR: 0.98, *P* value 0.020), while for gender it was found that, when correcting for age, BMI, musculoskeletal and other comorbidities, females have higher odds for completing the questionnaire as compared to males (OR: 1.50, *P* value; 0.010). BMI and having musculoskeletal or other comorbidities were not statistically significant associated with the completing of the GFI questionnaire for persons with end-stage hip or knee OA.

The internal consistency of the GFI in patients scheduled to undergo arthroplasty was 0.69, just below the threshold of 0.7 of good internal consistency [31]. Regarding the validity of the GFI questionnaire the mental and physical domains of GFI were strongly to moderately correlated with the MCS of the SF-12 (*R* = − 0.59, *P* < 0.001) and the PCS (*R* = − 0.39, *P* < 0.001), respectively, confirming the validity of the questionnaire. When performing cross-over analysis by correlating the mental domain of the GFI to the PCS of the SF-12 discriminatory validity was confirmed with a very weak correlation (*R* = − 0.08; *P* < 0.001). In addition, the correlation of the physical domain of the GFI and MCS had a low correlation of *R* = − 0.28 (*P* < 0.001). The correlation of the GFI with the HOOS/KOOS-pain score was, as hypothesized, low and also confirmed its discriminatory value to distinguish between pain and frailty (*R* = − 0.23, *P* < 0.001).

Of the 2957 patients with end-stage hip or knee OA who did complete the questionnaire, 853 (28.8%) were considered frail (a score of ≥ 4 on GFI). Patients with hip OA scored on average higher on the GFI [mean (SD) score: 2.78 (2.41) versus 2.28 (1.99)] and were more often considered frail as compared to persons with knee OA (33.3 versus 24.1%). Table 4 shows that frail persons were statistically significantly more often female, older and had a higher BMI as compared to those who are not frail. Also, frail persons scored statistically significantly lower on all scales of physical functioning of the HOOS/KOOS as well as on the physical and mental component scale of the SF-12 before arthroplasty surgery.

**Table 4:** Comparison of demographic characteristics of frail and non-frail end stage OA-patients

			Frailty as measured by GFI				
			Non-frail		Frail		*P* value
Affected joint	Hip	*N* (%)	1018	(66.7%)	509	(33.3%)	< 0.001
	Knee	*N* (%)	1086	(75.9%)	344	(24.1%)	
Sex	Female	*N* (%)	1216	(58.4%)	620	(73.6%)	< 0.001
BMI		Mean (SD)	28.07	(4.41)	28.69	(5.14)	0.002
Age	Years	Mean (SD)	67.07	(9.02)	68.99	(9.97)	< 0.001
HOOS/KOOS	Pain	Mean (SD)	40.56	(17.53)	32.96	(18.45)	< 0.001
	Symptoms	Mean (SD)	43.05	(16.25)	38.19	(16.34)	< 0.001
	Activities of daily life	Mean (SD)	45.35	(18.22)	34.97	(18.51)	< 0.001
	Sport	Mean (SD)	16.07	(17.54)	10.64	(14.57)	< 0.001
	Quality of Life	Mean (SD)	34.49	(10.79)	31.06	(9.75)	< 0.001
SF-12	PCS	Mean (SD)	33.38	(9.52)	29.33	(7.80)	< 0.001
	MCS	Mean (SD)	58.33	(6.79)	47.01	(11.06)	< 0.001
Comorbidities	Musculoskeletal	*N* (%)	351	(17.5%)	234	(29.4%)	< 0.001
	Other	*N* (%)	1248	(68.1%)	594	(84.4%)	< 0.001

*BMI* body mass index, *HOOS/KOOS* hip disability/knee injury and osteoarthritis and outcome score, *SF-12* short form 12 questionnaire, *PCS* physical component scale of the SF-12, *MCS* mental component scale of the SF-12

Differences between persons who are frail and those who are not. Frail and non-frail groups were compared by means of a *t* test (continue, normally distributed variable), Mann–Whitney (continue, not normally distributed variable) or Chi square (discrete variable), whichever was appropriate. A score of ≥ 4 was considered frail

## Discussion

The GFI is a valid questionnaire to assess frailty in end-stage hip or knee OA patients by means of a self-reported postal questionnaire. According to the GFI, using the cut-off of 4, about one-third of the patients undergoing THA and a quarter of the persons undergoing TKA are frail.

The feasibility of the use of the GFI within the current clinical setting for patients with end-stage hip or knee OA is good, as 90% of the participants completed the questionnaire. In a study by Metzelthin et al. in older community dwelling persons showed that 77.4% of the persons completed the questionnaire [32].

Those who did not complete the questionnaire were more often male and older. The open question (question 15) was most often left empty, indicating that it is probably easier for patients to have closed questions with predefined answer options. Further research is needed to reconsider the format of this question aiming to obtain higher response rates.

Although the Cronbach’s alpha of 0.69 is just below the threshold of good internal consistency of 0.7, it does indicate that the internal consistency of the GFI in our patient group is satisfactory and it is comparable to the alpha of 0.68 as found by Peters et al. in home dwelling elderly in the Netherlands [20, 31].

With respect to the convergent and discriminatory validity of the GFI for this specific patient group, the magnitude of the observed associations was in line with our hypotheses. Our convergent validity (range − 0.6–0.4) was comparable to the findings of Peters et al. (range 0.4–0.61) [20]. The discriminatory validity in our patient group (range − 0.08 to − 0.3) was even stronger as compared to the elderly of Peters et al. (range 0.08–0.5) [20].

Significantly more patients with end-stage hip OA were considered to be frail as compared to end-stage knee OA (hip; 33%, knee; 24%, *P* < 0.001). However, both these numbers are lower as compared to the study of Peters et al. who found 60% of the independent living elderly in their study to be frail as measured by the GFI, but the average age in that study was 81 years, much higher than in the present study (mean age 68 years) [20]. In a study among Romanian home-dwelling elderly (mean age 75), 75% of the participants were considered frail by the GFI [33]. These studies show that the presence of frailty shows wide variability depending on country, social status, diagnosis and age. The median and mean scores of the GFI in our patient group (2.00 and 2.54, respectively) were lower than the averages in independent living old persons found by Peters et al. (median 3) or reported by Metzelhin et al. and Drubbel et al. (means 3.8 and 3.2, respectively) [20, 32, 34]. In both the latter studies the mean age was higher than in our study (77 and 73 years, respectively). The lower frailty score in our patient groups can, apart from age, be explained by the fact that all patients were selected by an orthopaedic surgeon to receive arthroplasty surgery and were thus considered to be fit enough for major surgery.

The rates of persons with OA classified as being frail in our study are not easy to compare with other studies, as different methods to ascertain frailty were employed. Using Fried’s Frailty Phenotype [6], Mandl et al. found that 8% of persons scheduled for knee arthroplasty were considered frail (although 17% reported difficulty with activities of daily life) [35], with a similar rate found in men with hip osteoarthritis (8%) [36] and in a study of persons with knee, hip or hand OA from six different European cohorts (10.2% considered frail) [37].

A larger proportion, i.e. 22.4% of persons with hip or knee OA, was considered frail using Fried’s Frailty Phenotype in a Brazilian study [38]. With the interpretation of these proportions it must be taken into account that the criteria of Fried’s Frailty Phenotype [6] are to be ascertained by a physician and do not include activities of daily life.

Dent et al. have published an overview of the most commonly used frailty-questionnaires including, besides the GFI, three other self-reported frailty assessments: the Tilburg Frailty Index, the PRISMA-7 and the SPQ [39]. However, none of these other three self-reported questionnaires have to our knowledge been used to assess the occurrence of frailty in persons with osteoarthritis.

Since a large proportion, about one-third of the patients scheduled to undergo major implant surgery are considered frail as scored by the self-reported GFI, the effects of frailty on their postoperative outcome should be assessed in future studies. This study has shown that the use of the GFI to discriminate between frail and non-frail total joint arthroplasty patients is appropriate.
